# Glycoprotein IIb/IIIa and P2Y_12_ Induction by Oligochitosan Accelerates Platelet Aggregation

**DOI:** 10.1155/2014/653149

**Published:** 2014-08-28

**Authors:** Mercy Halleluyah Periayah, Ahmad Sukari Halim, Nik Soriani Yaacob, Arman Zaharil Mat Saad, Abdul Rahim Hussein, Ahmad Hazri Abdul Rashid, Zanariah Ujang

**Affiliations:** ^1^Reconstructive Sciences Unit, School of Medical Sciences, Universiti Sains Malaysia, 16150 Kubang Kerian, Kelantan, Malaysia; ^2^Department of Chemical Pathology, School of Medical Sciences, Universiti Sains Malaysia, 16150 Kubang Kerian, Kelantan, Malaysia; ^3^Advanced Medical & Dental Institute, Universiti Sains Malaysia, No. 1-8, Persiaran, Seksyen 4/1, Bandar Putra Bertam, 13200 Kepala Batas, Pulau Pinang, Malaysia; ^4^Industrial Biotechnology Research Centre, SIRIM Berhad, No. 1 Persiaran Dato' Menteri, Section 2, P.O. Box 7035, 40700 Shah Alam, Selangor, Malaysia

## Abstract

Platelet membrane receptor glycoprotein IIb/IIIa (gpiibiiia) is a receptor detected on platelets. Adenosine diphosphate (ADP) activates gpiibiiia and P2Y_12_, causing platelet aggregation and thrombus stabilization during blood loss. Chitosan biomaterials were found to promote surface induced hemostasis and were capable of activating blood coagulation cascades by enhancing platelet aggregation. Our current findings show that the activation of the gpiibiiia complex and the major ADP receptor P2Y_12_ is required for platelet aggregation to reach hemostasis following the adherence of various concentrations of chitosan biomaterials [7% N,O-carboxymethylchitosan (NO-CMC) with 0.45 mL collagen, 8% NO-CMC, oligochitosan (O-C), and oligochitosan 53 (O-C 53)]. We studied gpiibiiia and P2Y_12_ through flow cytometric analysis and western blotting techniques. The highest expression of gpiibiiia was observed with Lyostypt (74.3 ± 7.82%), followed by O-C (65.5 ± 7.17%). Lyostypt and O-C resulted in gpiibiiia expression increases of 29.2% and 13.9%, respectively, compared with blood alone. Western blot analysis revealed that only O-C 53 upregulated the expression of P2Y_12_ (1.12 ± 0.03-fold) compared with blood alone. Our findings suggest that the regulation of gpiibiiia and P2Y_12_ levels could be clinically useful to activate platelets to reach hemostasis. Further, we show that the novel oligochitosan is able to induce the increased expression of gpiibiiia and P2Y_12_, thus accelerating platelet aggregation *in vitro*.

## 1. Introduction

Platelets, which circulate within the blood, are the essential mediators that trigger the mechanical pathway of the coagulation cascade upon encountering any damage to the blood vessels. Platelets encourage primary hemostasis via three major processes: activation, adhesion, and aggregation. When the integrity of the vascular endothelium is interrupted, various macromolecular elements of the vascular subendothelium become exposed and readily accessible to platelets [1]. Platelet membrane receptor glycoprotein IIb/IIIa (gpiibiiia), also known as *α*_IIb_ *β*_3_, is an integrin complex and fibrinogen receptor that is detected on platelets. This integrin complex is essential for platelet aggregation and for platelet adherence to the endothelium, organized through the calcium-dependent association of gpIIb and gpIIIa [2–4]. Upon receptor activation, adenosine diphosphate (ADP) plays a vital role in modulating platelet function. The induction of platelet aggregation through the effective stimulation of 2 major ADP receptors (P2Y_1_ and P2Y_12_) has been well documented [5, 6]. In blood loss phenomena, ADP activates gpiibiiia and P2Y_12_, causing platelet aggregation and thrombus stabilization by recruiting more circulating platelets to the vessel injury to regulate adhesion. Biologically active hemostatic agents and sealants are used to stop hemorrhage by promoting hemostasis at sites of vascular injury. There is a large volume of published studies describing the role of hemostatic agents in assisting platelet mediators to expedite the mechanisms of hemostasis. In the new global economy, debate continues regarding the best strategies for the management of hemorrhage within the needed period of time. Chitosan biomaterials, which are extracted from marine arthropods, were found to promote surface-induced hemostasis and to be capable of activating blood coagulation cascades. Chitosan biomaterials have mainly been noticed because they function independently on platelets rather than abiding by normal clotting pathways. Chitosan is composed of an aminopolysaccharide molecule that possesses a strong positive charge, strongly attracting and bonding to negatively charged molecules [7–9]. However, the scientific evidence from the last decades on the mechanical pathways of platelet-specific behavior upon the adherence of chitosan-derived biomaterials remains controversial and unclear. Many studies have emphasized the prominent benefit of chitosan biomaterials, elucidating promising outcomes towards efficiently altering platelet activities. In contrast, no research thus far has reviewed the role of gpiibiiia and P2Y_12_ mediators in assisting platelet adhesion and aggregation. As a consequence, we have designed a study to determine the significant involvement of chitosan biomaterials on the expression of these mediators in expediting the process of hemostasis. In our present work, we tested N,O-carboxymethylchitosan (NO-CMC) and oligochitosan (O-C), chitosans produced by the Standard and Industrial Research Institute of Malaysia (SIRIM Berhad) with a degree of deacetylation of 75–98%. Our results demonstrated the ability of chitosan-based hemostatic agents to induce the potentiality of intracellular signaling cascades that involve the release of gpiibiiia and P2Y_12_ upon the possible stimulation of platelet activity by chitosan bioadhesives.

## 2. Materials and Methods

### 2.1. Biomaterials

Chitosan sponges with variable chitosan formulations (7% NO-CMC with 0.45 mL collagen, 8% NO-CMC, O-C, and a powdered variety of chitosans termed O-C 53) were used. Lyostypt was used as the positive control.

### 2.2. Reagents

Antibodies (Ab)s for flow cytometry were purchased from Becton Dickinson ((BD), NJ, USA): anti-CD 61 (integrin beta 3 chain), PerCP (clone RUU-PL 7F12 hybridization of mouse P3X63.Ag8.653 myeloma cells with spleen cells from BALB/c mice immunized with purified platelet membrane glycoproteins) (CD61 PerCP (340506)), and a fluorescein isothiocyanate- (FITC-) conjugated monoclonal antibody to CD41a (clone HIP8, derived from hybridization of mouse P3X63 myeloma cells with spleen cells from BALB/c mice immunized with purified platelet membrane glycoprotein) (CD41a FITC (340929)). All Abs for western blotting were purchased from Abcam (England, UK): (primary antibodies (1°): anti-P2Y_12_ Ab (rabbit polyclonal to P2Y_12_) (ab82725), dilution 1 : 1000; anti-beta actin (rabbit polyclonal to beta-actin-loading control) (ab8227), dilution 1 : 2000; and secondary antibody (2°): chicken polyclonal secondary Ab to rabbit IgG-H&L (HRP) (ab6829), dilution 1 : 2000). The Aurum serum protein mini kit and protein standards and ladder for SDS-PAGE (Precision Plus Protein Western Standards Value Pack) were purchased from Biorad (USA). The enhanced chemiluminescence detection kit (Amersham ECL Select western blotting detection reagent) was purchased from GE Healthcare life sciences (UK).

### 2.3. Flow Cytometry

#### 2.3.1. Blood Collection

Twelve milliliters of whole blood was drawn by venipuncture from 14 healthy donors into 4.5 mL Vacutainer tubes containing 3.2% buffered sodium citrate anticoagulant (REF 363083) using a butterfly needle. Blood was obtained with written informed consent. Prior to commencing the study, ethical clearance was obtained from the Human Ethics Committee of Universiti Sains Malaysia (USM). None of the female donors were taking oral contraceptives at the time of collection. None of the healthy donors had been diagnosed with chronic diseases. Subject selection was contingent upon a hematocrit level between 38% and 45% and a normal platelet count between 150 × 10^3^/*μ*L and 350 × 10^3^/*μ*L. To evaluate gpiibiiia expression, three-way stopcocks were used to collect blood under minimal tourniquet pressure, and the first 1 mL of blood withdrawn was discarded to minimize tissue factor-induced activation. The remainder of each blood sample was aliquoted into 3 tubes containing 3.2% sodium citrate.

#### 2.3.2. Sample Preparation

Freshly drawn blood was subjected to centrifugation at 100 g at room temperature (20–25°C) for 15 min [10]. Chitosan samples, each weighing 10 mg, were dissolved in 50 *μ*L of phosphate-buffered saline (PBS) (pH 7.4) and subjected to incubation at 37°C for 60 min. One hundred microliters of platelet-rich plasma (PRP) was then mixed with each prepared chitosan sample for 30 min [11–13]. Ten microliters of PRP was aliquoted in two different FACS tubes; the first tube contained unstained cells, and the second tube was incubated with CD61 PerCP (340506) and CD41a FITC (340929) (BD, USA). Stained and unstained cells were incubated in a dark at room temperature for 10 min. The reaction was stopped with 500 *μ*L of cold FACS buffer. Samples were analyzed immediately or kept at 4°C and analyzed within 24 hours. The expression of gpiibiiia was analyzed using flow cytometry.

#### 2.3.3. Analysis of Platelets with Flow Cytometry

Flow cytometric measurements of platelets were performed with the FACSCanto II (Becton-Dickinson Bioscience, USA) flow cytometer. The process of sample analysis prior to flow cytometric analysis is fundamentally comprised of five major steps: (1) chitosan-adhered blood sample preparation; (2) flow cytometer system and reagent setup; (3) cell fixation process; (4) cell permeabilization and immunolabeling; and (5) fluorescent staining and platelet counterstaining. A polygonal gate was created to propose each dot plot pattern. Four regions were proposed and labeled Q1, Q2, Q3, and Q4. The quadrants represent positivity for the following markers: Q1: CD 41a FITC-A; Q2: CD 41a FITC-A and CD61 PerCP-A; Q3: unstained/instrument noise; and Q4: CD61 PerCP-A within the gpiibiiia-positive population. At least 10,000 events were analyzed to calculate the mean fluorescence intensity (MFI) (%) of the total platelet population. The detectors were set to logarithmic amplification. The acquisition and data interpretation were accomplished using BD FACS Diva software.

### 2.4. Western Blotting

#### 2.4.1. Blood Sample Collection and Preparation

Blood sample collection procedures were carried out as described above. The Aurum serum protein mini kit (Bio-Rad, USA) was used to maximize the resolution of major ADP receptor P2Y_12_ expression using western blotting techniques. Sample preparation consists of 3 major steps: (1) column setup; (2) sample binding and purification; and (3) collection of purified samples. Purified chitosan-treated samples were then subjected to a protein quantification test.

#### 2.4.2. Protein Quantification

Two microliters of purified chitosan-treated samples was added to measure protein quantity (mg/mL). Protein concentrations were determined using a NanoDrop 2000 UV-Vis Spectrophotometer (Thermo Fisher Scientific) at 280 nm absorbance.

#### 2.4.3. Western Blot Analysis

One hundred nanograms of protein was resolved on a 10% SDS-Polyacrylamide gel for 45 min followed by semidry transfer onto a Nitrocellulose membrane using the Trans-Blot SD semidry transfer cell (Bio-Rad, USA). The membrane was then blocked with 5% nonfat milk diluted in PBS-Tween 20 for 2 hours at RT under mild agitation. The membrane was then washed with 1*X* PBS for 10 min × 4 times and incubated with the 1° Ab (P2Y_12_ or *β*-actin Ab) in blocking buffer overnight at 4°C. On the following day, the incubated membrane was washed with PBS-Tween 20 (0.001%) for 10 min × 4 times. The washed membrane was then incubated with the 2° Ab conjugated to horseradish peroxidase in blocking buffer for 2 hours at RT. Then, the membrane was again washed with PBS-Tween 20 for 10 min × 4 times. The membrane was developed with the enhanced chemiluminescence detection system using FluorChem FC2 (USA), and analyses were conducted using AlphaView Software. The band density for each test was compared to the control—the bands were analyzed using ImageJ 1.46 software (provided by NIH (http://imagej.nih.gov/ij/)), and values were normalized to the *β*-actin band density [14, 15].

### 2.5. Statistical Analysis

The data are presented as percentages for gpiibiiia analysis (*n* = 14). Three independent experiments were carried out to demonstrate the expression of P2Y_12_. An independent *t*-test was applied to elucidate the mean ± standard error of mean (SEM) and the significance value of the expression levels. Statistical significance was defined as *P* ≤ 0.05, and these values were calculated using SPSS software, version 18.0.

## 3. Results

### 3.1. Expression of gpiibiiia

**Figure 1:**
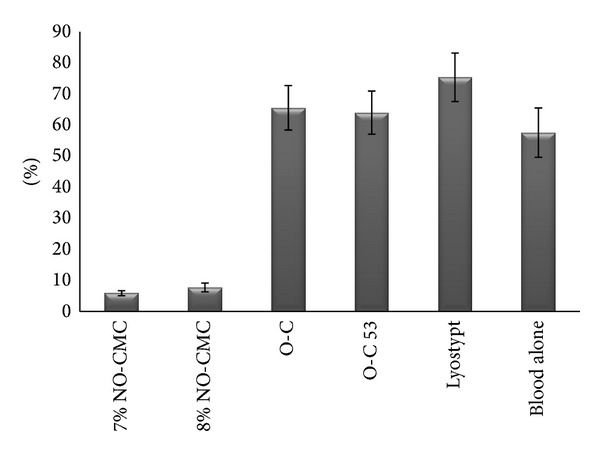
*In vitro* expression levels of gpiibiiia upon the adherence of chitosan biomaterial. Depicted is the average of the mean ± SEM of fourteen fluorescence measurements, as obtained by flow cytometry.

The mean fluorescence intensity of the total platelet populations was calculated from at least 10,000 events, in percentages. A polygonal gate was created to propose each dot plot pattern. Four regions were proposed and labeled Q1, Q2, Q3, and Q4. Each quadrant represents the following: Q1: CD 41a FITC-A; positive for gpiib; Q2: CD 41a FITC-A and CD61 PerCP-A; positive for gpiibiiia; Q3: unstained/instrument noise; and Q4: CD61 PerCP-A; positive for gpiiia. The percentage of platelets expressing gpiibiiia upon flow cytometric analysis ranged from 0.5% to 99.3%. The highest expression of gpiibiiia was observed with Lyostypt (74.3 ± 7.82%), followed by O-C (65.5 ± 7.17%). Lyostypt and O-C resulted in increases of 29.2% and 13.9%, respectively, compared with blood alone (*P* < 0.05). On the contrary, gpiibiiia was expressed at the lowest relative levels upon the adherence of two different types of NO-CMCs, which were 5.90 ± 0.77% for 7% NO-CMC and 7.74 ± 1.40% for 8% NO-CMC. O-C induced either a slight increase or equivalent expression levels compared with the blood alone. Chitosan stimulation of gpiibiiia expression varied between each donor (*n* = 14) (*P* < 0.05) (Figure 1).

**Figure 2:**
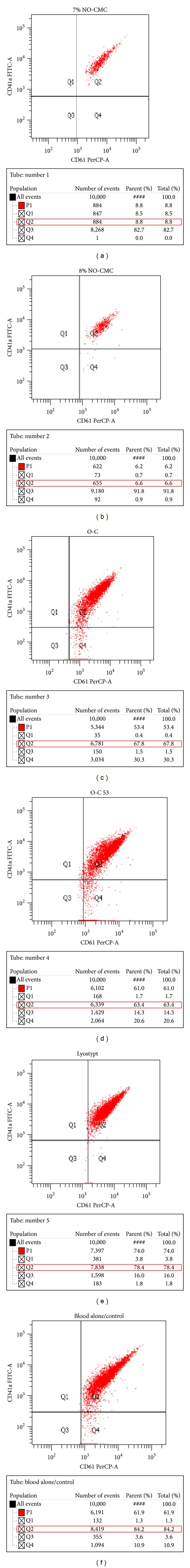
Example of gpiibiiia expression levels demonstrated after the adherence of chitosan biomaterials. The expression levels of gpiibiiia are depicted in the dot plot results (CD 41a FITC-A × CD 61 PerCP-A), and the gpiibiiia-positive population is expressed in Q2 region. The events and percentages of each tested biomaterial are indicated by the red boxes. The same gating and parameter settings (FITC × PerCP) were used for all tests (*n* = 14). (a) 7% NO-CMC; (b) 8% NO-CMC; (c) O-C; (d) O-C 53; (e) Lyostypt; and (f) blood alone.

The example of the dot plot results for the tested biomaterials presented in Figure 2 (CD 41a FITC-A × CD 61 PerCP-A) shows the population positive for gpiibiiia. The Q2 region is positive for gpiibiiia expression (values are presented as percentages and highlighted in red). As mentioned, the expression levels following the adherence of 7% NO-CMC and 8% NO-CMC correlated with the low percentages. The O-C chitosan group expressed gpiibiiia at equivalent levels but 6-fold greater percentages than NO-CMCs (Figures 2(a)–2(d)). As for the overall outcome, O-C induced the expression of gpiibiiia and functioned equivalently to blood alone (Figure 2(f)). Therefore, O-C was considered to work effectively as a bioadhesive material to induce gpiibiiia expression and to promote platelet aggregation to achieve hemostasis* in vitro*.

### 3.2. P2Y_12_ Expression

We have investigated the expression of P2Y_12_ receptor signaling upon the adherence of bioadhesive chitosan biomaterials, which aid in platelet adhesion and thrombus formation. Western blot analysis (*n* = 3) revealed that O-C 53 was capable of upregulating the expression of P2Y_12_  1.12 ± 0.03-fold when compared with blood alone. Additionally, Lyostypt, the commercially available hemostatic agent used as positive control, was capable of inducing the upregulation of P2Y_12_ by 1.11 ± 0.04-fold. On the contrary, the remaining chitosan biomaterials tested downregulated the expression of P2Y_12_, as follows: O-C with a 0.94 ± 0.05-fold change; 7% NO-CMC with a 0.87 ± 0.04-fold change; and 8% NO-CMC with a 0.87 ± 0.06-fold change. The dotted line in Figure 2 depicts the expression level in the blood alone, which we fixed to 1 when calculating the band densities in ImageJ. Although the fold changes observed are very close for each biomaterial, O-C 53 exclusively induced the highest fold-change in expression.

## 4. Discussion

Platelets are the first-line fundamental elements that support the maintenance of hemostasis. This mechanical system arrests blood loss whenever there is an injury to the vessel wall. Hemostasis is triggered by 3 major pathogenic events: platelet activation, adhesion, and aggregation [16]. Platelet aggregation requires the engagement of platelets via physical forces once platelets are activated by the adherence of any antihemorrhagic biomaterials. However, fibrin formation occurs due to chemical synthesis of fibrin polypeptides through the actions of enzymes and cofactors. Platelet aggregation is mediated by the gpiibiiia receptor, which was discovered to be one of the abundant cell surface receptors (80 × 10^3^ per platelet) and represents 15% of total surface proteins [17, 18]. Over the past years, ADP and gpiibiiia receptors have been targeted for antithrombotic strategies with drug compounds, such as ticlopidine, clopidogrel, abciximab, eptifibatide, and tirofiban, as alternative therapies to reduce vascular complications [19]. Chitosan-derived hemostatic agents are capable of activating platelets by providing a good surface for blood coagulation mediators and signals to facilitate thrombin generation, which is then able to stimulate gpiibiiia activation. Thrombin is one of the blood coagulation factors, and the presence of thrombin suggests the existence of clots. A large number of researchers have successfully investigated chitosan biomaterials in blood coagulation, either by chitosan alone or fabricated with other naturally obtained polymers, due to their prominent biodegradable, biocompatible, nontoxic, and antihemorrhagic properties. Studies have been proposed that chitin and chitosan have specific properties based on their solubility in aqueous acetic acid, as chitosan is soluble, but chitin is insoluble. Thermal intervention and modification of chitin under firm aqueous alkali are normally required to provide partially deacetylated chitin (30–90%), which is then considered chitosan. Deacetylation (DA) is a process that releases the amine groups (NH2), generating chitosan with cationic characteristics. The percentage of DA was given more careful thought while preparing chitosan due to the influence of the acidic environment, whereas the majority of polysaccharides appears to be neutral or negatively charged [20–22]. Although the importance of chitin was first described in 1811 by Henri Braconnot and has been highlighted over the years, chitosan has recently taken a central role in solving many medical difficulties in the new global economy [23].

Our present paper generated information regarding the activation of the gpiibiiia complex and ADP major receptor P2Y_12_, required for platelet aggregation to complete the hemostasis process, upon the adherence of various concentrations of chitosan biomaterials produced by SIRIM Berhad, with DA values <85% and molecular weights <40000 Da. Additionally, no studies thus far have reviewed the role of gpiibiiia and P2Y_12_ mediators in assisting platelet adhesion and aggregation in the presence of chitosan-derived biomaterials. We have utilized two different markers (CD 41a FITC-A and CD 61 PerCP-A) to detect the expression of gpiibiiia on platelets. CD41/CD61 is a complex composed of noncovalently associated CD41 and CD61. This complex is derived from a member of the integrin family primarily expressed on platelets and megakaryocytes. CD41/CD61 mediates platelet aggregation and platelet adhesion to the extracellular matrix [24–26]. Chitosan stimulation of gpiibiiia expression varies between individuals. Both 7% and 8% NO-CMCs induced low levels of gpiibiiia expression. The O-C group of chitosan resulted in a 6-fold greater percentage of platelets expressing gpiibiiia than NO-CMCs. As for the overall outcome, O-C induced gpiibiiia expression and function equal to blood alone and was considered to work effectively as a bioadhesive material to induce gpiibiiia expression and to promote platelet aggregation and hemostasis* in vitro*. Platelet gpiibiiia is a calcium-dependent receptor that acts with adhesive proteins on the membrane surface of platelets, with the assistance of O-C, to mediate platelet binding to a variety of cells and substrate.

We used 7% NO-CMC that was coated and premixed with 0.45 mL of bovine collagen in predetermined compositions and freeze-dried to obtain a porous structure. The main reason for using collagen-coated chitosan in our study was that collagen is capable of initiating platelet aggregation at sites of tissue injury [7]. Collagen is one of the most abundant proteins and can be extracted from nearly every single living animal. The common resources for the collagen used in tissue engineering and regenerative applications are mainly bovine skin and tendons. Collagen proteins have been shown to play essential roles in platelet adhesion and the acceleration of platelet activation to trigger the increased production of procoagulant phospholipids [27]. Nevertheless, 7% NO-CMC was unable to produce an effective increase in gpiibiiia expression. However, our previous results suggested that 7% NO-CMC was able to recruit platelets and enhance platelet aggregation on porous scaffolds [28]. The exact mechanism that controls the expression of activated gpiibiiia complexes remains unknown [29, 30].

The P2Y_12_ receptor is a member of the G protein-coupled receptor family and is triggered by ADP to activate various signaling pathways involved in the amplification of platelet activation and aggregation [31]. As highlighted in the results section, we successfully studied the expression of P2Y_12_ in the presence of two different classes of chitosan biomaterials via western blotting. Western blot analysis of band densities revealed that O-C 53 upregulated the expression of P2Y_12_ by 1.12-fold. Moreover, *β*-actin expression was not influenced or affected by the adherence of chitosan biomaterials to blood proteins throughout the experiments. Although chitosan biomaterials are thought to work in mysterious ways, as the exact mechanical functions have not been revealed, the hemostatic properties of chitosan biomaterials are known to entirely rely on several important aspects, including the molecular weight, degree of deacetylation, solubility, chitosan concentration, positive charge density, chemical structure modification, pH, temperature, incubation period, and hydrophilic and hydrophobic characteristics [32]. However, controversy still exists over the scientific evidence into the mechanical pathways of platelet-specific behavior following the adherence of chitosan-derived biomaterials. Therefore, further study with more focus on the technical aspects of O-C is suggested to show a strong relationship between O-C and platelet mechanisms.

## 5. Conclusions

Our findings suggest that the analysis of gpiibiiia and P2Y_12_ is clinically useful for platelet activation to reach hemostasis. In the present study, we showed that different chitosan groups with different formulations differentially activate platelet mechanisms. A novel oligochitosan, either in sponge or in powdered form, was able to induce expression levels of gpiibiiia and P2Y_12_ to accelerate platelet aggregation* in vitro*. The value “53” added to O-C is just a sequence identification figure to distinguish between the sponge and powdered type of chitosans. Despite the considerable amount of work carried out to emphasize the importance of gpiibiiia and P2Y_12_ expression in the presence of chitosan-derived biomaterials, the mechanical pathways of both platelet activities and chitosans remain unclear. A better understanding of how platelet signals are coordinated during stimulation with chitosan biomaterials is necessary to clarify the mechanism of chitosan-mediated hemostasis.

## Figures and Tables

**Figure 3:**
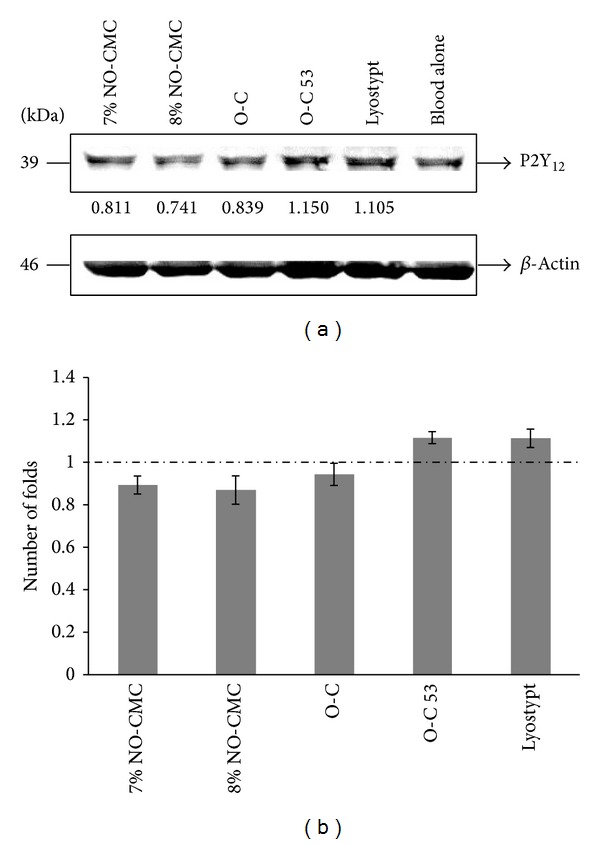
Expression of P2Y_12_ upon the adherence of chitosan biomaterials* in vitro*. Protein was extracted from PRP exposed to chitosan, and the expression of P2Y_12_ was determined by western blotting (Figure 3(a)). The protein bands were visualized using an image analyzer and band density was analyzed using ImageJ software, with the fold-change differences in expression compared with blood alone written below each band.  Figure 3(b). The expression levels of P2Y_12_ upon the presence of chitosan biomaterials are shown as the fold change in comparison with blood alone (1-fold). The blots shown are representative of the mean ± SEM from three independent experiments (*n* = 3). The dotted line depicts the expression level in the blood alone (1-fold).
